# Cumulative exposures to glycaemia and lipids are associated with coronary artery disease in type 1 diabetes: a call for action

**DOI:** 10.1186/s12933-025-02803-8

**Published:** 2025-06-13

**Authors:** Rebecka Bergdal, Valma Harjutsalo, Per-Henrik Groop, Stefan Mutter, on behalf of the FinnDiane Study Group

**Affiliations:** 1 Folkhälsan Research Center, Biomedicum Helsinki, Helsinki, Finland; 2 Department of Nephrology, University of Helsinki and Helsinki University Hospital, Helsinki, Finland; 3 Research Program for Clinical and Molecular Metabolism, Faculty of Medicine, University of Helsinki, Helsinki, Finland; 4 Department of Diabetes, Central Clinical School, Monash University, Melbourne, VIC Australia; 5 Baker Heart and Diabetes Institute, Melbourne, VIC Australia

**Keywords:** Coronary artery disease, Cumulative glycaemic exposure, Cumulative lipid exposure, Type 1 diabetes mellitus

## Abstract

**Background:**

Hyperglycaemia and dyslipidaemia are well-known risk factors for coronary artery disease (CAD) in type 1 diabetes. The impact of long-term cumulative exposure to these risk factors is less explored. We investigated the relationship between cumulative glycaemic and lipid exposure and CAD in individuals with type 1 diabetes.

**Methods:**

This longitudinal study included 3495 adults with type 1 diabetes from the FinnDiane cohort, without end-stage kidney disease and no history of CAD or stroke at the study baseline. Total cumulative glycaemic exposure (CGE_tot_) and cumulative hyperglycaemic exposure (CGE_hg_), accounting only for time spent above an HbA_1c_ of 53 mmol/mol (7%), were calculated from diabetes diagnosis.

**Results:**

During a median follow-up of 19.38 years, 534 participants had their first-ever CAD event. CGE_hg_ (odds ratio 1.03 [95% CI 1.02–1.05], p < 0.001) and cumulative exposure to LDL cholesterol, triglycerides, and non-HDL cholesterol all significantly increased the odds for incident CAD. The highest tertile of CGE_hg_ associated with a twofold odds increase for incident CAD. CGE_tot_ was not significantly associated with CAD after adjusting for cumulative lipid exposure.

**Conclusions:**

Both hyperglycaemia and dyslipidaemia are independently associated with CAD in type 1 diabetes. These findings emphasize the importance of reaching an HbA_1c_ below 53 mmol/mol (7%) and minimizing lipid exposure, as well as calling on health care professionals to not settle for suboptimal care, but to continue their support and encouragement towards better management of diabetes.

**Graphical Abstract:**

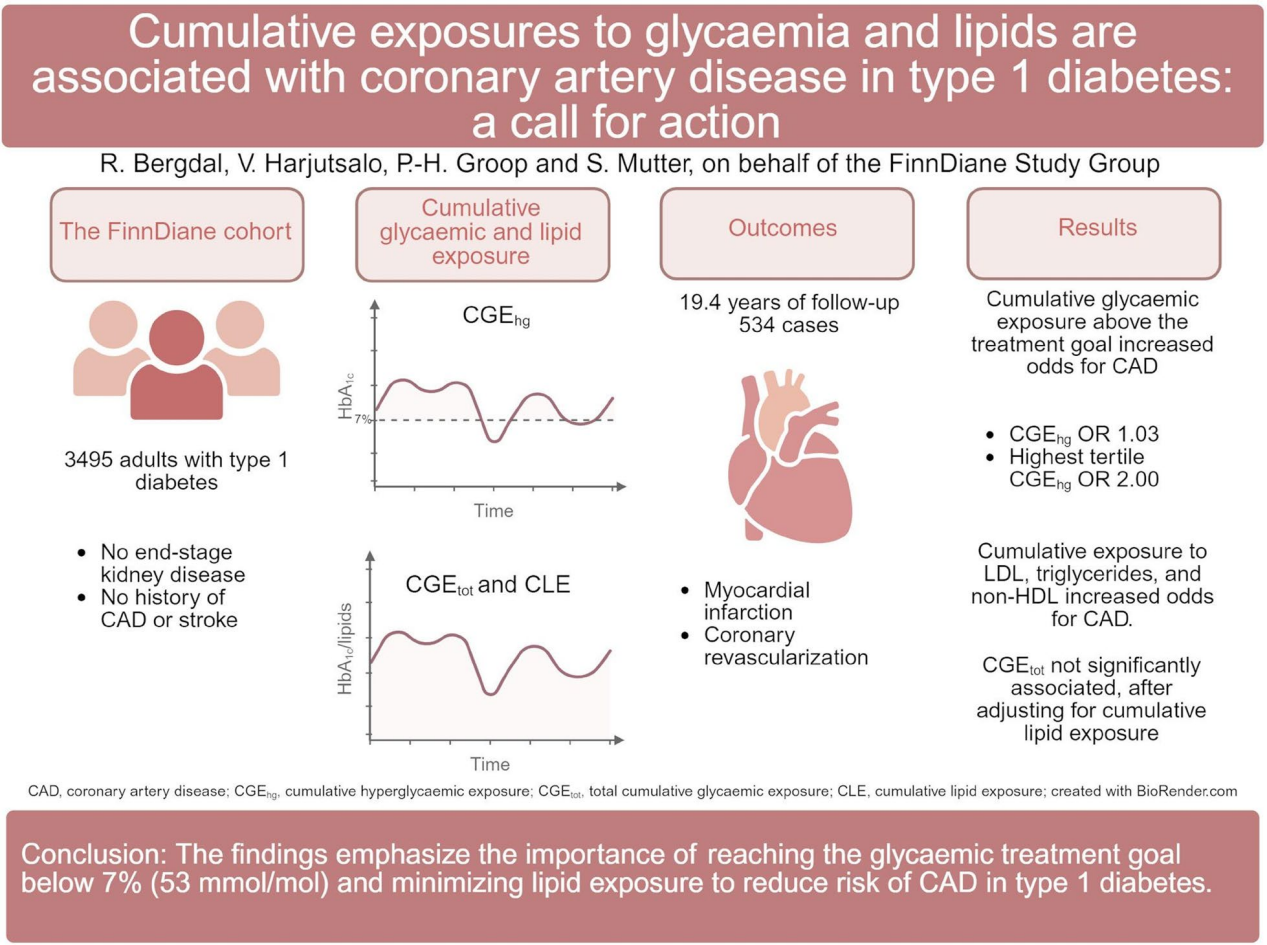

**Supplementary Information:**

The online version contains supplementary material available at 10.1186/s12933-025-02803-8.

## Research insights

**What is currently known about this topic?**

- Cumulative glycaemic exposure is associated with increased risk of microvascular complications in type 1 diabetes. There is less evidence regarding the association with macrovascular complications.

**What is the key research question?**

- How does cumulative glycaemic exposure relate to risk of coronary artery disease (CAD) in type 1 diabetes and how is the relationship affected by cumulative lipid exposure?

**What is new?**

- Cumulative glycaemic exposure above the treatment goal increases the odds for CAD in type 1 diabetes. Also, cumulative exposure to LDL cholesterol, triglycerides, and non-HDL cholesterol independently increased the odds for incident CAD.

**How might this study influence clinical practice?**

- The findings emphasize the importance of reaching the glycaemic treatment goal and minimizing lipid exposure to reduce CAD risk in type 1 diabetes.

## Background

The risk of cardiovascular disease (CVD) and cardiovascular mortality is substantially higher among individuals with type 1 diabetes than in the general population [1, 2]. Glycaemic control, variability of HbA_1c_, and diabetes duration are all well-known risk factors for diabetes complications [3–5]. The VISS study investigated the threshold HbA_1c_ for development of different microvascular complications in type 1 diabetes. With longer diabetes duration the threshold has been lowered for all complications, suggesting an effect of cumulative glycaemic exposure [6]. Importantly, studies have shown that different measures of cumulative glycaemic exposure are better predictors of microvascular complications than single HbA_1c_ measurements or mean HbA_1c_ [7–9]. In individuals with type 1 diabetes, the association between cumulative glycaemic exposure and macrovascular complications such as coronary artery disease (CAD) has been investigated only in smaller cohort studies. In the Epidemiology of Complications (EDC) study cohort (N = 434), increased cumulative glycaemic exposure was associated with increased risk of diabetes complications, including CAD [9].

Furthermore, the Diabetes Control and Complications Trial and its follow-up study the Epidemiology of Diabetes Interventions and Complications study (DCCT/EDIC) found a prolonged risk-reducing effect of early good glycaemic control, called “*metabolic memory*”[10]. They demonstrated that participants initially selected into the intensive treatment group had a 30% reduced risk of cardiovascular events, and an even greater reduction in risk of severe events even 20 years after the end of the trial, compared to participants receiving standard treatment during the DCCT [11]. There are several different hypotheses regarding the mechanisms behind metabolic memory and, importantly for this study, there has been debate concerning whether metabolic memory could be solely explained by cumulative glycaemic exposure [9, 10].

However, hyperglycaemia is only one of many identified risk factors for CVD. In individuals with type 1 diabetes, kidney disease is one of the most important predictors of CVD together with hyperglycaemia [12]. Also, traditional risk factors such as dyslipidaemia and hypertension must be considered when assessing the risk of CAD in diabetes. In the general population LDL cholesterol is not only a risk factor but evidently a causal factor for atherosclerotic cardiovascular disease [13]. The evidence for LDL as a predictor of CAD in type 1 diabetes is, however, conflicting [4, 14, 15]. Instead, triglycerides appear to have a strong influence on CAD risk in type 1 diabetes [4, 16]. The evidence regarding associations between lipids and CAD in type 1 diabetes is mainly derived from analyses that include single lipid measurements or long-term means. The impact of cumulative lipid exposure has not previously been investigated, nor have the effects of cumulative glycaemic and cumulative lipid exposures together in relation to CAD incidence in type 1 diabetes.

Thus, the aims of this study were to investigate the association between cumulative glycaemic exposure and CAD in a large cohort of Finnish adults with type 1 diabetes with a long follow-up. Additionally, we investigated the relationship between cumulative glycaemic and cumulative lipid exposures. Lastly, in a subcohort of participants we examined the association between cumulative glycaemic exposure before baseline and CAD, to explore the relationship between cumulative glycaemic exposure and metabolic memory and shed further light into their roles as predictors of CAD in the Finnish Diabetic Nephropathy (FinnDiane) study cohort.

## Methods

### Cohort description and selection

**Fig. 1:**
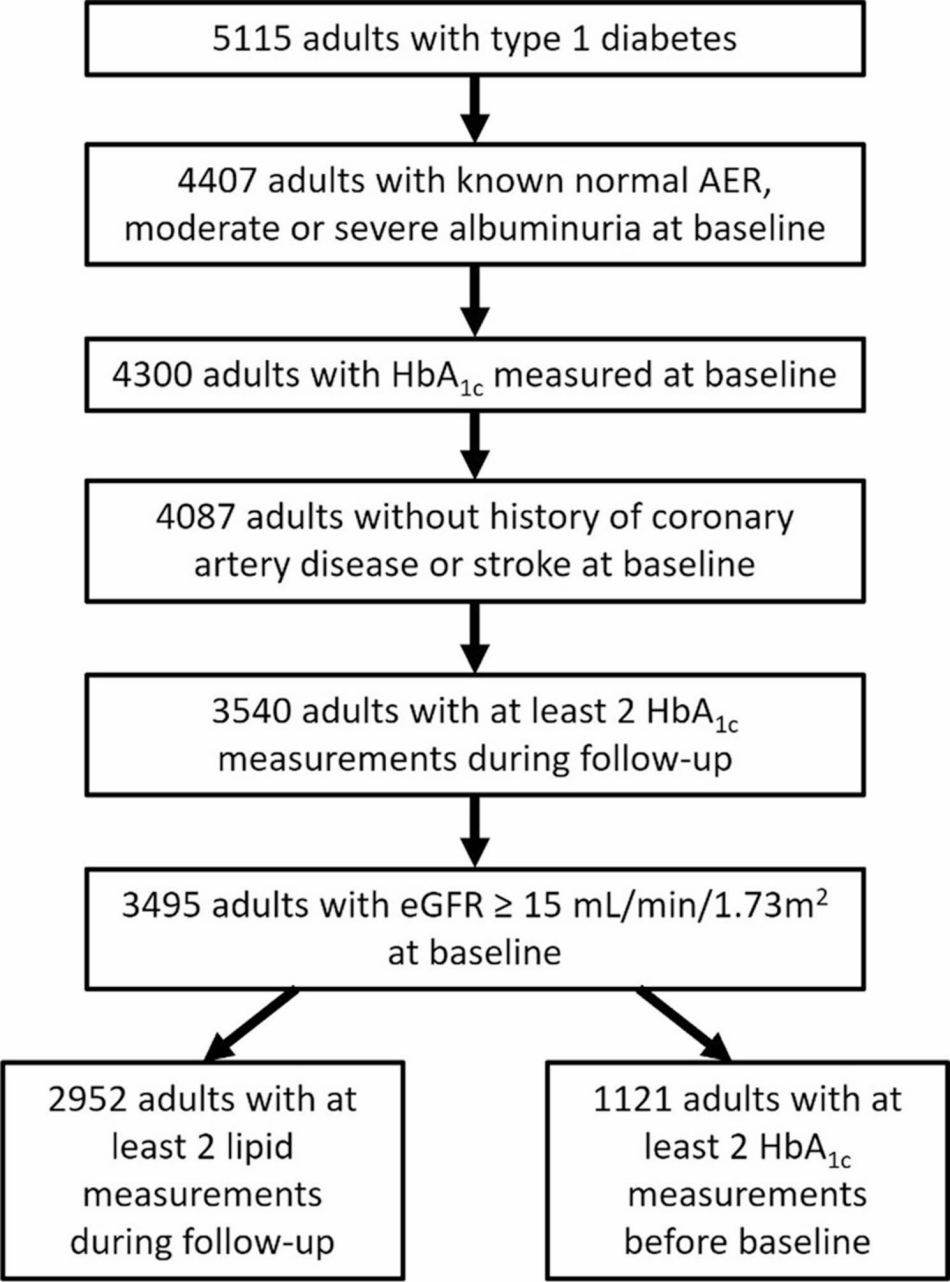
Cohort selection

This study included 3495 adults from the FinnDiane study, which is a nationwide, multi-centre, prospective cohort of currently over 5100 adults with type 1 diabetes that was launched in 1997, with recruitment of new participants still ongoing. The cohort selection for this specific study is illustrated in Fig. 1. Those excluded from the study had a worse cardiovascular risk profile, but importantly no difference in baseline HbA_1c_ (Additional file 1: Table S1). Type 1 diabetes was defined as diabetes onset below 40 years of age and requiring insulin within one year of diagnosis. Participants underwent a thorough clinical investigation at the baseline study visits. During the visit blood pressure, body weight, height, and waist and hip circumference were measured, as well as fasting blood and urine samples were collected. Data on medication, cardiovascular status, and diabetic complications including acute myocardial infarction, stroke and coronary heart disease were registered by a standardized questionnaire, which was completed by the participant’s attending physician based upon the medical file or a study nurse in our Helsinki study centre. In addition, serum measurements such as HbA_1c_ and lipids were collected from the participants’ medical records before and after their baseline visit and from follow-up study visits if available. For this study, individuals with end-stage kidney disease, or history of CAD or stroke at baseline were excluded. The data was gathered from the Care Register for Health Care. Those with no HbA_1c_ measurement at baseline or less than two HbA_1c_ measurements during follow-up were also excluded. It is important to note that the time between two consecutive measurements is not based on regular intervals and depends on data availability.

### Outcome measures

The primary outcome was first-ever CAD event, defined as first instance of nonfatal myocardial infarction, coronary revascularization, or CAD death. Data on the primary outcomes were obtained from the Care Register for Health Care (ICD-10: I21, I22, I23; ICD-8 and ICD-9: 410, 412; procedure codes for coronary revascularization). Moreover, fatal events were verified from the Causes of Death Register. The follow-up ended at first-ever CAD event, death, or censoring at the end of year 2020, whichever occurred first.

### Cumulative glycaemic exposure and cumulative lipid exposure

We hypothesized that all exposure to glycaemia and lipids during the whole diabetes duration have an impact on the risk of complication development. Therefore, the cumulative glycaemic and lipid exposures for each participant were calculated from diabetes diagnosis until the first-ever CAD event, death, or censoring at the end of year 2020, whichever occurred first. Two different measures were calculated to estimate cumulative glycaemic exposure using HbA_1c_ measurements (in percentage units rather than in mmol/mol) during follow-up. The method used was adapted from a metric introduced by the EDC study called “A1c Months” [9]. The first measure, called cumulative hyperglycaemic exposure (CGE_hg_), takes only the time spent above the HbA_1c_ treatment goal of 53 mmol/mol (7%) into account [17]. CGE_hg_ was calculated by adding two components: the cumulative hyperglycaemic exposure prior to the first available HbA_1c_ measurement and the cumulative hyperglycaemic exposure afterwards. Cumulative hyperglycaemic exposure prior to the first measurement was estimated by multiplying the HbA_1c_ units above 53 mmol/mol (7%) at the first available measurement by the number of months from diabetes diagnosis to the time of the first measurement. For each measurement afterwards, the HbA_1c_ units above 53 mmol/mol (7%) were multiplied by the number of months between the midpoints of the measurement and the measurements directly before and after. The products were then summed to obtain the cumulative hyperglycaemic exposure during follow-up. The second measure, called total cumulative glycaemic exposure (CGE_tot_), was calculated using the same method as for CGE_hg_, only without any threshold for HbA_1c_. A schematic illustration of the calculation method for cumulative glycaemic measures is shown in the additional file (Additional file 1: Figure S1). Further, the same method was used to estimate total cumulative lipid exposure (without any threshold) for participants with lipid data available (N = 2952). The exposure to LDL cholesterol (CLE_LDL_), triglycerides (CLE_tg_), and non-HDL cholesterol (CLE_non-HDL_) were calculated separately.

### Statistical analyses

Continuous variables are reported as mean and standard deviation if normally distributed and as median with interquartile range otherwise. Categorical variables are presented as percentages. To analyse differences between groups Student t-test or ANOVA were used for continuous variables, which were normally distributed and Mann–Whitney U or Kruskal–Wallis test otherwise. Pearson’s chi-squared test was used for categorical variables. Logistic regression models were used to assess the association between cumulative glycaemic exposure and CAD. The first model included the measure for cumulative glycaemic exposure adjusted for unmodifiable risk factors, including sex and age at diabetes onset. Further, the models were adjusted for cumulative lipid exposure, first for CLE_LDL_, CLE_tg_, and CLE_non-HDL_ separately and finally, adjusting for both CLE_LDL_ and CLE_tg_.

As the time from diagnosis to first HbA_1c_ measurement was longer among those with incident CAD during follow-up, a sensitivity analysis was performed with a dataset matched by the time from diagnosis until first HbA_1c_ measurement. Each individual with incident CAD (534 cases) were matched with two controls (1068 controls). The same logistic regression models were applied.

In a subcohort (N = 1121) with at least two HbA_1c_ measurements prior to baseline, we employed survival models. They allowed us to investigate metabolic memory by examining how cumulative glycaemic exposure from diabetes diagnosis to the baseline of the study affected the incidence of CAD after baseline. Cox proportional hazards models with non-CAD death as a competing risk were used to analyse this association. The risk of CAD was evaluated at five years, ten years, and full follow-up. Three models were used: (1) adjusted for unmodifiable risk factors age, sex, and diabetes duration at baseline; (2) further adjusted for non-HDL cholesterol and triglycerides at baseline; (3) additionally adjusted for lipid-lowering medication, hypertension, BMI, and kidney disease according to KDIGO class which takes both albuminuria and estimated glomerular filtration rate into account [18].

To facilitate the interpretation of the results for metabolic memory, the change in glycaemic control in the cohort over time was investigated. A weighted mean HbA_1c_ was calculated by dividing cumulative glycaemic exposure over a certain time by that time in months. The time spans examined were from diagnosis to baseline, from baseline to five years, from baseline to ten years and from baseline to the end of follow-up. The change in glycaemic control was compared between individuals with and without incident CAD.

Details of the statistical methods are described in the supplementary material (Additional file 1). All analyses were performed in R version 4.4.2 (2024–10-31) and the Cox regression was based on the survival package [19, 20]. P-values < 0.05 were considered statistically significant.

## Results

### Characteristics at the study baseline visit

The clinical baseline characteristics are presented in Table 1 according to incident CAD. Out of 3495 participants, 534 (15%) had their first ever CAD event during a median follow-up of 19.38 (15.40, 21.47) years (60 433 person-years). Individuals with incident CAD had a worse clinical risk profile already at baseline. HbA_1c_ was significantly higher, they were older, had a longer duration of diabetes, higher BMI, higher blood pressure, lower eGFR and were more likely to have albuminuria. Antihypertensive and lipid-lowering medications were more common among those with incident CAD. Their lipid profiles were more atherogenic, including lower HDL cholesterol and higher LDL, triglycerides, and non-HDL. There was no significant difference in sex or age at diabetes onset.

**Table 1:** Baseline characteristics according to incident first-ever CAD event during follow-up

Variable	N	CAD −, N = 2961^a^	CAD +, N = 534^a^	p-value
Men (%)	3495	50	54	0.088
Age (years)	3495	35.5 ± 10.9	44.7 ± 10.8	< 0.001
Age at onset (years)	3495	16.4 ± 9.5	16.1 ± 9.5	0.4
Duration of diabetes (years)	3495	19.0 ± 11.1	28.7 ± 11.0	< 0.001
BMI (kg/m2)	3463	25.1 ± 3.6	25.8 ± 3.6	< 0.001
Waist/Hip-ratio	3386	0.86 ± 0.08	0.89 ± 0.09	< 0.001
Waist/Height-ratio	3384	0.50 ± 0.06	0.52 ± 0.06	< 0.001
SBP (mmHg)	3461	131 ± 16	141 ± 18	< 0.001
DBP (mmHg)	3460	79 ± 10	81 ± 10	0.001
HbA_1c_ (mmol/mol)	3495	67.8 ± 16.2	72.7 ± 15.4	< 0.001
HbA_1c_ (%)	3495	8.4 ± 1.5	8.8 ± 1.4	< 0.001
Antihypertensive medication (%)	3479	28	58	< 0.001
Renin-angiotensin system (RAS) inhibitors (%)	3480	26	50	< 0.001
Lipid-lowering medication (%)	3481	7.5	20	< 0.001
Total cholesterol (mmol/L)	3480	4.8 ± 0.9	5.3 ± 1.0	< 0.001
LDL-C (mmol/L)	3480	3.0 ± 0.8	3.4 ± 0.9	< 0.001
HDL-C (mmol/L	3480	1.4 ± 0.4	1.3 ± 0.4	< 0.001
Triglycerides (mmol/L)	3480	1.0 (0.7, 1.4)	1.2 (0.9, 1.7)	< 0.001
ApoB (g/L)	3426	84.5 ± 22.1	96.1 ± 22.7	< 0.001
Non–HDL-C (mmol/L)	3480	3.5 ± 0.9	4.0 ± 1.1	< 0.001
eGFR (mL/min/1.73 m2)	3495	101.7 ± 22.9	84.5 ± 28.6	< 0.001
eGDR (mg/kg/min)	3376	6.9 (4.8, 8.8)	4.8 (3.5, 6.7)	< 0.001
Normal AER (%)	3495	75	49	< 0.001
Moderate albuminuria (%)	3495	14	18	0.006
Severe albuminuria (%)	3495	11	33	< 0.001
Ever smoker (%)	3340	45	50	0.031
Retinal laser treatment (%)	3475	23	53	< 0.001
Pump-users (%)	3471	5.6	3.0	0.016

^a^%; Mean ± SD; Median (IQR)

### Glycaemic exposure from diabetes diagnosis

The cumulative glycaemic exposure from diabetes diagnosis until end of follow-up was significantly higher among individuals with incident CAD, both when examining the total exposure CGE_tot_ and when only considering the time spent above an HbA_1c_ of 53 mmol/mol (7%), CGE_hg_ (Table 2). All cumulative lipid measures were higher in this group as well. However, the diabetes duration at the end of follow-up was also longer in those with a CAD event. But all cumulative exposures per month were higher in those with incident CAD (Table 2).

**Table 2:** Cumulative glycaemic exposure and cumulative lipid exposure according to incident first-ever CAD event during follow-up

Variable	N	CAD−, N = 2961^a^	CAD +, N = 534^a^	p-value
Diabetes duration at end of follow-up	3495	37.20 ± 11.52	41.03 ± 10.91	< 0.001
Follow-up time (years)	3495	19.86 (17.06, 21.82)	13.10 (7.83, 17.02)	< 0.001
Time from diagnosis to first HbA_1c_ measurement (years)	3495	15.94 (8.31, 24.05)	26.48 (18.92, 34.54)	< 0.001
Number of HbA_1c_ measurements	3495	26.00 (15.00, 40.00)	24.00 (13.00, 38.75)	0.045
CGE_tot_	3495	3748.09 ± 1238.31	4349.04 ± 1193.63	< 0.001
CGE_hg_	3495	565.75 (269.02, 932.21)	843.57 (509.16, 1268.72)	< 0.001
CGE_tot_/month	3495	8.41 ± 1.17	8.90 ± 1.28	< 0.001
CGE_hg_/month	3495	1.32 (0.67, 2.08)	1.77 (1.09, 2.63)	< 0.001
Time from diagnosis to first lipid measurement (years)	2952	16.85 (8.92, 25.07)	27.31 (20.47, 34.90)	< 0.001
Number of lipid measurements	2952	10.00 (6.00, 16.00)	10.00 (7.00, 15.00)	0.7
CLE_LDL_	2952	1243.73 ± 514.21	1582.28 ± 550.96	< 0.001
CLE_tg_	2952	427.93 (309.73, 598.96)	565.67 (403.58, 824.12)	< 0.001
CLE_non-HDL_	2952	1451.61 ± 581.19	1859.71 ± 625.30	< 0.001
CLE_LDL_/month	2952	2.74 ± 0.65	3.18 ± 0.76	< 0.001
CLE_tg_/month	2952	0.96 (0.75, 1.29)	1.16 (0.87, 1.60)	< 0.001
CLE_non-HDL_/month	2952	3.21 ± 0.75	3.76 ± 0.91	< 0.001

^a^Median (IQR); Mean ± SD

### Glycaemic exposure and incident CAD

Odds for incident CAD were increased with both higher CGE_tot_ and CGE_hg_ when adjusted for unmodifiable risk factors sex and age at onset (Table 3). The association between CGE_hg_ and CAD remained significant when adjusted for cumulative lipid exposure, whereas CGE_tot_ was no longer significantly associated with CAD when adjusted for CLE_non-HDL_ or for both CLE_LDL_ and CLE_tg_. All cumulative lipid measures were associated with increased odds for incident CAD. Participants were further divided into tertiles according to CGE_hg_. With the lowest tertile as reference, the highest tertile of CGE_hg_ was associated with increased odds for incident CAD in the fully adjusted model.

**Table 3:** Logistic regression models assessing association of cumulative glycaemic exposure and cumulative lipid exposure with CAD

Logistic regression model	Variable	OR (CI 95%)^a^	p-value
Model 1	CGE_tot_	1.05 (1.04–1.06)	< 0.001
Model 2	CGE_tot_	1.02 (1.00–1.03)	0.016
	CLE_LDL_	1.10 (1.07–1.13)	< 0.001
Model 3	CGE_tot_	1.03 (1.02–1.04)	< 0.001
	CLE_tg_	2.35 (1.84–3.01)	< 0.001
Model 4	CGE_tot_	1.01 (1.00–1.02)	0.144
	CLE_non-HDL_	1.11 (1.08–1.14)	< 0.001
Model 5	CGE_tot_	1.01 (0.99–1.02)	0.279
	CLE_LDL_	1.08 (1.05–1.11)	< 0.001
	CLE_tg_	2.01 (1.55–2.60)	< 0.001
Logistic regression model	Variable	OR (CI 95%)	p-value
Model 1	CGE_hg_	1.05 (1.04–1.06)	< 0.001
Model 2	CGE_hg_	1.04 (1.03–1.05)	< 0.001
	CLE_LDL_	1.11 (1.09–1.13)	< 0.001
Model 3	CGE_hg_	1.04 (1.03–1.05)	< 0.001
	CLE_tg_	2.49 (1.98–3.14)	< 0.001
Model 4	CGE_hg_	1.04 (1.03–1.05)	< 0.001
	CLE_non-HDL_	1.11 (1.08–1.13)	< 0.001
Model 5	CGE_hg_	1.03 (1.02–1.05)	< 0.001
	CLE_LDL_	1.09 (1.06–1.11)	< 0.001
	CLE_tg_	1.71 (1.31–2.22)	< 0.001
Logistic regression model	Variable	OR (CI 95%)	p-value
Model 1	CGE_hg_:		
	< 395 (Reference)	1.0	
	395–841	1.71 (1.32–2.23)	< 0.001
	> 841	3.10 (2.43–3.95)	< 0.001
Model 2	CGE_hg_:		
	< 395 (Reference)	1.0	
	395–841	1.45 (1.07–1.96)	0.016
	> 841	2.34 (1.74–3.13)	< 0.001
	CLE_LDL_	1.11 (1.09–1.14)	< 0.001
Model 3	CGE_hg_:		
	< 395 (Reference)	1.0	
	395–841	1.38 (1.02–1.86)	0.038
	> 841	2.22 (1.65–2.99)	< 0.001
	CLE_tg_	2.62 (2.08–3.29)	< 0.001
Model 4	CGE_hg_:		
	< 395 (Reference)	1.0	
	395–841	1.41 (1.04–1.90)	0.028
	> 841	2.18 (1.62–2.92)	< 0.001
	CLE_non-HDL_	1.11 (1.09–1.13)	< 0.001
Model 5	CGE_hg_:		
	< 395 (Reference)	1.0	
	395–841	1.33 (0.98–1.81)	0.064
	> 841	2.00 (1.48–2.70)	< 0.001
	CLE_LDL_	1.09 (1.06–1.11)	< 0.001
	CLE_tg_	1.79 (1.38–2.32)	< 0.001

Model 1 adjusted for unmodifiable risk factors sex and age at onset; model 2 adjusted for unmodifiable risk factors and cumulative LDL exposure; model 3 adjusted for unmodifiable risk factors and cumulative triglyceride exposure: model 4 adjusted for unmodifiable risk factors and cumulative non-HDL exposure; model 5 adjusted for unmodifiable risk factors, cumulative LDL exposure, and cumulative triglyceride exposure

^a^Per 100-units, except for CGE_hg_ and CLE_tg_ which are reported by square root and logarithmically, respectively

When participants with incident CAD were divided into tertiles based on diabetes duration at the end of follow-up, the mean cumulative glycaemic exposure and mean cumulative lipid exposure at CAD development increased with longer diabetes duration (Table 4). However, those with shorter diabetes duration at CAD development had a higher glycaemic and triglyceride exposure per month of follow-up, whereas exposure to LDL and non-HDL per month was the same regardless of diabetes duration.

**Table 4:** Cumulative glycaemic exposure and cumulative lipid exposure at CAD event and average CGE_tot_, CGE_hg_, CLE_LDL_, CLE_tg_, and CLE_non-HDL_ per month from diagnosis until CAD event, by tertiles of diabetes duration at event

	Diabetes duration at CAD event			p-value
	< 37 years^a^	37***–***45.4 years^a^	> 45.4 years^a^	
CGE_tot_	3202.7 ± 865.2	4430.7 ± 619.2	5413.8 ± 830.7	< 0.001
CGE_hg_	663.1 (372.5, 1044.0)	879.6 (615.6, 1281.0)	981.9 (591.6, 1446.6)	< 0.001
CGE_tot_/month	9.19 ± 1.47	8.95 ± 1.20	8.57 ± 1.05	< 0.001
CGE_hg_/month	1.93 (1.14, 3.09)	1.80 (1.23, 2.62)	1.52 (0.87, 2.29)	< 0.001
CLE_LDL_	1132.4 ± 389.2	1579.0 ± 386.3	1992.5 ± 492.6	< 0.001
CLE_tg_	454.6 (320.5, 649.3)	573.7 (440.8, 825.3)	686.1 (523.9, 884.7)	< 0.001
CLE_non-HDL_	1360.7 ± 470.0	1861.3 ± 455.1	2309.6 ± 547.5	< 0.001
CLE_LDL_/month	3.21 ± 0.80	3.19 ± 0.78	3.15 ± 0.70	0.827
CLE_tg_/month	1.32 (0.93, 1.89)	1.17 (0.89, 1.67)	1.07 (0.84, 1.45)	0.001
CLE_non-HDL_/month	3.86 ± 0.99	3.76 ± 0.92	3.66 ± 0.82	0.186

^a^Median (IQR); Mean ± SD

A significant part of the cumulative glycaemic exposure was estimated based on the first available HbA_1c_ measurement, which covered around 40% of the variation seen in the cumulative glycaemic exposure before baseline (adjusted R2: 0.39). As the time from diagnosis until first HbA_1c_ measurement was longer in individuals with incident CAD, a sensitivity analysis was conducted with 1602 individuals (534 cases) matched by the time from diagnosis to first recorded HbA_1c_ measurement (Additional file 1: Table S2) which confirmed the results. In the matched dataset, the follow-up time was significantly longer among individuals without incident CAD (19.61 [16.30, 21.61] vs 13.10 [7.83, 17.02]) (Additional file 1: Table S3), resulting in higher CGE_tot_ (4523.38 ± 1182.57 vs 4349.04 ± 1193.63) in individuals without incident CAD, whereas CGE_hg_ still remained lower (667.53 [334.92, 1038.94] vs 843.57 [509.16, 1268.72]). Therefore, the association between CGE_hg_ and CAD remained significant, whereas CGE_tot_ was inversely associated with CAD due to survival bias (Table 5). CLE_tg_ was the only lipid measure that remained significant in all models in the sensitivity analysis.

**Table 5:** Logistic regression models assessing association of cumulative glycaemic exposure and cumulative lipid exposure with CAD, matched dataset

Logistic regression model	Variable	OR (CI 95%)^a^	p-value
Model 1	CGE_tot_	0.99 (0.98–1.00)	0.282
Model 2	CGE_tot_	0.98 (0.97–1.00)	0.012
	CLE_LDL_	1.05 (1.02–1.08)	0.002
Model 3	CGE_tot_	0.98 (0.97–0.99)	0.001
	CLE_tg_	2.34 (1.75–3.12)	< 0.001
Model 4	CGE_tot_	0.98 (0.96–0.99)	< 0.001
	CLE_non-HDL_	1.06 (1.03–1.09)	< 0.001
Model 5	CGE_tot_	0.97 (0.96–0.99)	< 0.001
	CLE_LDL_	1.03 (1.00–1.06)	0.077
	CLE_tg_	2.19 (1.63–2.95)	< 0.001
Logistic regression model	Variable	OR (CI 95%)^a^	p-value
Model 1	CGE_hg_	1.04 (1.03–1.05)	< 0.001
Model 2	CGE_hg_	1.04 (1.03–1.05)	< 0.001
	CLE_LDL_	1.01 (0.99–1.03)	0.427
Model 3	CGE_hg_	1.03 (1.02–1.05)	< 0.001
	CLE_tg_	1.51 (1.15–1.99)	0.003
Model 4	CGE_hg_	1.04 (1.03–1.05)	< 0.001
	CLE_non-HDL_	1.02 (0.99–1.04)	0.136
Model 5	CGE_hg_	1.03 (1.02–1.05)	< 0.001
	CLE_LDL_	0.99 (0.97–1.02)	0.652
	CLE_tg_	1.55 (1.15–2.10)	0.004
Logistic regression model	Variable	OR (CI 95%)^a^	p-value
Model 1	CGE_hg_:		
	< 395 (Reference)	1.0	
	395–841	1.50 (1.12–2.02)	0.007
	> 841	2.32 (1.75–3.08)	< 0.001
Model 2	CGE_hg_:		
	< 395 (Reference)	1.0	
	395–841	1.49 (1.07–2.07)	0.018
	> 841	2.36 (1.72–3.25)	< 0.001
	CLE_LDL_	1.01 (0.99–1.03)	0.364
Model 3	CGE_hg_:		
	< 395 (Reference)	1.0	
	395–841	1.41 (1.01–1.97)	0.044
	> 841	2.08 (1.51–2.88)	< 0.001
	CLE_tg_	1.60 (1.22–2.09)	< 0.001
Model 4	CGE_hg_:		
	< 395 (Reference)	1.0	
	395–841	1.47 (1.06–2.05)	0.022
	> 841	2.29 (1.67–3.16)	< 0.001
	CLE_non-HDL_	1.02 (1.00–1.04)	0.093
Model 5	CGE_hg_:		
	< 395 (Reference)	1.0	
	395–841	1.41 (1.01–1.97)	0.043
	> 841	2.10 (1.51–2.90)	< 0.001
	CLE_LDL_	0.99 (0.97–1.02)	0.597
	CLE_tg_	1.65 (1.23–2.21)	< 0.001

Model 1 adjusted for unmodifiable risk factors sex and age at onset; model 2 adjusted for unmodifiable risk factors and cumulative LDL-C exposure; model 3 adjusted for unmodifiable risk factors and cumulative triglyceride exposure: model 4 adjusted for unmodifiable risk factors and cumulative non-HDL-C exposure; model 5 adjusted for unmodifiable risk factors, cumulative LDL-C exposure, and cumulative triglyceride exposure

^a^Per 100-units, except for CGE_hg_ and CLE_tg_ which are reported by square root and logarithmically, respectively

### Metabolic memory

We tested for metabolic memory effects in a subcohort of 1121 adults with data on HbA_1c_ before baseline. Of those 174 (15%) had their first-ever CAD event during follow-up, 35 occurring within five years from baseline and 75 within ten years. The mean number of HbA_1c_ measurements before baseline was 16 measurements. Individuals with incident CAD had higher before baseline CGE_tot_ (3294.01 ± 1222.26 vs. 2197.17 ± 1146.54, p < 0.001) and CGE_hg_ (708.06 [325.22, 1164.97] vs. 312.00 [111.75, 609.12], p < 0.001). Before baseline CGE_hg_ was a significant predictor of a CAD event at both five years and ten years of follow-up (Table 6). Additionally, before baseline CGE_tot_ and CGE_hg_ were both significantly associated with future CAD when accounting for the full follow-up time of 17.20 (9.95, 20.03) years (Table 6). The association between before baseline CGE_hg_ and future CAD remained significant also after further adjustment for CGE_hg_/month after baseline (Additional file 1: Table S4). After baseline, median glycaemic control improved in those with incident CAD during follow-up and in the cohort as a whole (p-value < 0.001), and there was no significant difference in the rate of improvement between those with and without incident CAD (Additional file 1: Table S5 and S6).

**Table 6:** Competing risk regression examining the association between before baseline cumulative glycaemic exposure and first ever CAD event during follow-up

Competing risk model, 5 years, N = 35	Variable	HR (CI 95%)^a^	p-value
Model 1	CGE_tot_	1.08 (1.03–1.13)	0.003
Model 2	CGE_tot_	1.06 (1.01–1.11)	0.018
Model 3	CGE_tot_	1.05 (0.99–1.10)	0.088
Competing risk model, 5 years, N = 35	Variable	HR (CI 95%)	p-value
Model 1	CGE_hg_	1.06 (1.02–1.09)	0.001
Model 2	CGE_hg_	1.05 (1.01–1.08)	0.005
Model 3	CGE_hg_	1.04 (1.00–1.07)	0.035
Competing risk model, 10 years, N = 75	Variable	HR (CI 95%)	p-value
Model 1	CGE_tot_	1.06 (1.02–1.10)	0.003
Model 2	CGE_tot_	1.05 (1.01–1.09)	0.013
Model 3	CGE_tot_	1.03 (1.00–1.08)	0.086
Competing risk model, 10 years, N = 75	Variable	HR (CI 95%)	p-value
Model 1	CGE_hg_	1.04 (1.02–1.07)	< 0.001
Model 2	CGE_hg_	1.03 (1.01–1.06)	0.002
Model 3	CGE_hg_	1.03 (1.00–1.05)	0.024
Competing risk model, full follow-up, N = 174	Variable	HR (CI 95%)	p-value
Model 1	CGE_tot_	1.07 (1.04–1.10)	< 0.001
Model 2	CGE_tot_	1.06 (1.03–1.09)	< 0.001
Model 3	CGE_tot_	1.05 (1.02–1.08)	0.002
Competing risk model, full follow-up, N = 174	Variable	HR (CI 95%)	p-value
Model 1	CGE_hg_	1.04 (1.03–1.06)	< 0.001
Model 2	CGE_hg_	1.04 (1.02–1.05)	< 0.001
Model 3	CGE_hg_	1.03 (1.01–1.05)	< 0.001

Model 1 adjusted for unmodifiable risk factors sex, age, and diabetes duration; model 2 adjusted for unmodifiable risk factors and lipids: non-HDL cholesterol and triglycerides; model 3 adjusted for unmodifiable risk factors, lipids and hypertension, lipid-lowering medication, obesity, and kidney disease

^a^CGE_tot_ is reported per 100-units, CGE_hg_ by square root

A sensitivity analysis was conducted for the competing risk regression as well, including 522 individuals (174 cases) with HbA_1c_ measurements before baseline, matched by the time from diagnosis to first recorded HbA_1c_ measurement. The results were consistent with those of the unmatched analysis (Additional file 1: Table S7). Furthermore, when restricting the analysis to the 191 participants (9 cases, 182 controls) with first HbA_1c_ measurement within 5 years from diabetes diagnosis, both before baseline CGE_hg_ (HR 1.1 [1, 1.21], p = 0.041) and CGE_tot_ (HR 1.12 [1.04, 1.21], p = 0.005) were significantly associated with CAD in an unadjusted model.

## Discussion

### Cumulative glycaemic exposure

In this study, we have shown that cumulative glycaemic exposure increased the odds of the first ever CAD event in individuals with type 1 diabetes. Especially, the time spent above the recommended HbA_1c_ treatment goal of 53 mmol/mol (7%) was of importance, as this increased the odds of CAD even when adjusting for cumulative lipid exposure. We found that the individuals in the highest tertile of CGE_hg_ had a twofold odds increase for incident CAD during follow-up compared with those in the lowest tertile. Additionally, in the Cox regression analysis, the hazard ratio for CAD over the full-time was 1.03 (per square root of CGE_hg_ increase). These results are comparable to those in the smaller EDC cohort, where the hazard ratio for CAD in the A1c Month top quintile was 2.7 [9]. The CGE_hg_ in the lowest and highest tertile equalled the cumulative glycaemic exposure acquired over 40 years of diabetes with an HbA_1c_ below 62 mmol/mol (7.82%) and above 72 mmol/mol (8.75%), respectively, suggesting that a long-term reduction of HbA_1c_ by roughly one percentage point decreases the odds for CAD by half. The findings emphasize the importance of early and ongoing aggressive treatment of hyperglycaemia, to reach the treatment goal. The non-linear relationship between CGE_hg_ and CAD further suggests not settling with glycaemic control only close to the target. These results were confirmed in a sensitivity analysis only including participants with matched time from diagnosis to first HbA_1c_ measurement. The sensitivity analysis was conducted to exclude potential bias caused by estimating a longer period of glycaemic exposure based only on one measurement, seen as cumulative glycaemic exposure before the first available measurement was calculated based solely on the HbA_1c_ level at that first measurement. Additionally, the sensitivity analysis revealed the superiority of CGE_hg_ compared to CGE_tot_ and susceptibility of the latter to survival bias, as it continues to increase even with normal HbA_1c_ values. Due to longer follow-up time the CGE_tot_ was higher in those without incident CAD in the matched dataset, causing CGE_tot_ to associate inversely with CAD in this analysis. Therefore, this study showed that, especially, the cumulative glycaemic exposure above the treatment goal is a CAD risk indicator.

Although cumulative glycaemic exposure is a variable that describes long-term glycaemic control more comprehensively than single or mean HbA_1c_, it does not take into account all aspects of glycaemic control. The FinnDiane study have previously shown that HbA_1c_ variability also predicts CAD events, independently of mean HbA_1c_ [5]. CGE_hg_ accounts for variability around the treatment target of 7%, but not for variability at different HbA_1c_ levels, and CGE_tot_ does not account for variability at all. Neither does cumulative glycaemic exposure consider the impact of hypoglycaemia which is also associated with increased risk of cardiovascular disease [21]. This said, the results of this study and previous findings on HbA_1c_ variability and hypoglycaemia all point to the benefits of a good and stable glycaemic control.

When the risk of retinopathy in the DCCT cohort was assessed, total glycaemic exposure was an equally good predictor as cumulative hyperglycaemic exposure, regardless of threshold used (37–53 mmol/mol [5.5–7%]) [7]. However, in this study CGE_tot_ did not significantly increase the odds for CAD in the fully adjusted models in the main analysis. The contrary results might relate to differences in pathophysiology and the role of glycemia for the development of micro- and macrovascular complications. Hyperglycaemia accelerates the development of atherosclerosis, but lipids are causal factors [22]. This might explain why CGE_tot_ was no longer significant for incident CAD once the cumulative lipid exposure was taken into account. A recent study showed a significant association between total cumulative glycaemic exposure and CVD in type 2 diabetes also after adjusting for baseline lipids [23]. However, long-term lipid exposure was not considered, as it was in this study.

### Cumulative lipid exposure

To our knowledge, this is the first study to investigate the effect of both cumulative glycaemic and cumulative lipid exposure on the incidence of CAD in type 1 diabetes. CLE_LDL_, CLE_tg_, and CLE_non-HDL_ were all significantly associated with incident CAD. The role of LDL as a risk factor for CAD in type 1 diabetes is debated and the evidence is inconsistent [4, 14, 15]. A Norwegian cross-sectional study found an association of mean HbA_1c_ and LDL cholesterol with prevalent coronary artery disease in a cohort of long-term survivors of type 1 diabetes [24]. Of note, although the cohort was on average well controlled in terms of HbA_1c_ and other risk factors, an increased time-weighted mean HbA_1c_ increased the odds for CAD. In turn, lower mean LDL increased the odds for absence of coronary plaques as defined by Holte et. al. In a previous FinnDiane study, LDL was a significant predictor only in individuals with poor glycaemic control or macroalbuminuria, whereas non-HDL was more strongly associated with CAD in the full cohort [16]. In this study, both CLE_LDL_ and CLE_non-HDL_ were associated with CAD. Triglycerides are one of the lipid variables that are most strongly associated with CAD in type 1 diabetes [4, 16]. This study confirms previous findings and shows that the association also extends to cumulative triglyceride exposure, which was the lipid variable with the strongest association with CAD.

Poor glycaemic control is strongly associated with higher LDL, non-HDL, and triglycerides, which together lead to high risk of CAD [25, 26]. It is unclear whether the association reflects a common underlying factor or if poor glycaemic control causes dyslipidaemia. Insulin resistance, which is associated with both poor glycaemic control and a more atherogenic lipid profile in type 1 diabetes, has been suggested as a potential mediating factor [27, 28]. Current interventions in type 1 diabetes to reduce insulin resistance are mainly focussing on diet and exercise [29]. Other proposed pathways linking glycaemic control and dyslipidaemia are advanced glycation end-product formation and glycation of LDL particles, which by many different mechanisms promote atherosclerosis in diabetes and increase LDL atherogenicity [30]. Findings from the DCCT/EDIC cohort suggests that up to 50% of the increased risk of CVD caused by glycemia is mediated by the adverse risk profile, including dyslipidaemia, associated with poor glycaemic control [31]. Nevertheless, glycemia itself remains a significant risk factor. This is in line with the results of this study, where the increased odds of CAD related to long-term hyperglycaemia remained significant. There is further evidence suggesting that the effect of hyperglycaemia as a risk factor for CVD is enhanced by higher triglyceride concentrations [32]. In turn, the EDC study identified a subgroup of participants (20%) with an unexpected combination of high HbA_1c_ and low non-HDL showing only a minor increase in total mortality and CVD as underlying or contributing cause of death, compared to participants with low HbA_1c_ and low non-HDL [33]. Furthermore, in a study on a non-diabetic population with untreated familial hypercholesterolemia, LDL receptor mutations were associated with better glycaemic control, pointing to the influence of different genotypes on the relationship between glycaemic control and lipid metabolism. Despite better glycaemic control, subjects with LDL receptor mutations had higher prevalence of coronary artery calcification [34]. The FinnDiane study has previously identified rare gene variants in individuals with type 1 diabetes that are associated with different lipid, apolipoprotein, and lipoprotein phenotypes [35]. Another previous study in our cohort found only a modest genetic contribution towards HbA_1c_ [36]. However, studies in type 2 diabetes have shown that a higher genetic risk for type 2 diabetes was associated with poorer glycaemic control [37, 38]. Further studies focusing on different patterns and combinations of cumulative glycaemic and cumulative lipid exposure, as well as further exploration of the influence of genetic factors, are needed to gain a deeper understanding of the interaction between these risk factors for CAD in type 1 diabetes.

### Metabolic memory

In a subcohort of 1121 adults with HbA_1c_ data available before the FinnDiane study baseline, we found that higher cumulative glycaemic exposure before baseline predicted future CAD. Also, the DCCT/EDIC study reported that prior poor glycaemic control result in a prolonged risk-increasing effect on both microvascular and macrovascular complications, which they called metabolic memory [10]. On the other hand, it was observed in the EDC study that, at the time of CAD, there was no significant difference in average cumulative glycaemic exposure regardless of diabetes duration, suggesting that complications start to occur when reaching a certain amount of cumulative glycaemic exposure [9]. Contrary to the EDC results, in the FinnDiane cohort, the cumulative glycaemic exposure at CAD development increased by longer diabetes duration and so did the cumulative lipid exposures, while the mean exposures per month were higher with shorter diabetes duration. Further, before baseline CGE_hg_ remained significantly associated with future CAD even after adjusting for CGE/month after baseline. These findings might indicate that cumulative glycaemic exposure alone cannot explain all the risk increases related to hyperglycaemia, but that the pattern of the glycaemic exposure must be considered as well, thereby also supporting the concept of metabolic memory. In the DCCT/EDIC the metabolic memory effect started to fade 10 years after the end of DCCT. In contrast, in this study, before baseline cumulative glycaemic exposure remained a significant predictor of CAD throughout the 17 years of follow-up. Although the glycaemic control improved among those with incident CAD during follow-up, it remained poorer than for those without incident CAD. Therefore, the cumulative glycaemic exposure continued to increase more rapidly in the CAD group, potentially explaining why the higher risk remained. This higher risk also remained while standards of care improved over the study period.

Several different mechanisms have been suggested to explain the effects of metabolic memory and cumulative glycaemic exposure, the key mechanisms including oxidative stress, increased advanced glycation end-product formation and epigenetic modifications [39].

### Strengths and limitations

The main strength of this study is a large, well described cohort with a long follow-up. Also, both glycaemic and lipid exposure were thoroughly explored by accounting for both total glycaemic and hyperglycaemic exposure, and three different lipid measures including LDL, triglyceride, and non-HDL exposure separately. Several limitations must be acknowledged. The study was conducted in a Finnish population with a relatively homogeneous genetic and healthcare background, and the applicability of the results to other populations cannot be ensured. Due to the observational study setting, no conclusions on causality can be drawn. Furthermore, there were no predetermined intervals between HbA_1c_ measurements, leading to a large variation in the time between measurements, which in turn might reduce the accuracy of the glycaemic exposure calculations. Additionally, although the total cumulative exposures per month were higher in those adults with incident CAD, we cannot fully exclude a survival bias as diabetes durations at the end of follow-up were longer in those with incident CAD. It is important to note that a logistic regression model suffers from bias in risk assessment especially when timespans of accumulation differ between individuals and should be interpreted with caution. Nonetheless, the results from the logistic and the Cox regressions both support the conclusions. The Cox regression analysis, however, was employed only on a subset of participants, as information on HbA_1c_ measurements before baseline were not available for all. The analyses were not adjusted for all major risk factors for CAD, as information on some risk factors, such as hypoglycaemia, family history and smoking, were not available or missing for many participants. The use of lipid-lowering medication was considered, but no detailed information on the type of medication was available. The number of covariates was further limited by the small number of cases in the 5-year model. In addition, not all history of CVD was accounted for, as only participants with history of CAD or stroke were excluded, but not participants with history of heart failure (eight participants). There are also limitations to HbA_1c_ as an indicator of glycaemic control, e.g., a variety of average glucose levels and glucose patterns can result in the same HbA_1c_ levels [40]. Continuous glucose monitoring (CGM) overcomes this limitation and offers valuable additional insight into glucose patterns and variability. Further research evaluating cumulative glycaemic exposure based on complete data on glycaemic control from diabetes diagnosis onward and utilizing CGM is needed to validate the findings of this study and to more accurately evaluate the effect of cumulative glycaemic exposure on the development of complications in diabetes. New antihyperglycemic treatments including incretin-based therapies and sodium-dependent glucose transporter 2 inhibitors have improved treatment of type 2 diabetes, both by improving glycaemic control and reducing risk of cardiovascular events. Off-label use in individuals with type 1 diabetes have shown promising results, however, further studies on the efficacy and safety of these treatments in type 1 diabetes are required to confirm a potential benefit and before wider implementation into standard treatment.

## Conclusions

In conclusion, this study is the first to show cumulative lipid exposures independently increase odds for CAD in type 1 diabetes. While agreeing with previous research that hyperglycaemia and dyslipidaemia are associated with CAD in type 1 diabetes, this study highlights the importance of early and continuously minimizing the cumulative glycaemic and cumulative lipid exposures to reduce the risk of CAD. Although the quality of diabetes care and glycaemic control has improved, still a majority of individuals with type 1 diabetes do not reach the treatment target of HbA_1c_ below 53 mmol/mol (7%), reflecting the difficulty of achieving optimal care [41]. With this study and considering findings from previous studies, we call on health care professionals to support and encourage individuals with type 1 diabetes towards better management of type 1 diabetes, including both glucose and lipid management. Early and continuously achieving glycaemic targets remains a cornerstone to prevent complications of diabetes including CAD. Moreover, given the complexity of the mechanisms underlying diabetic complications, additional risk factors must not be overlooked.

## Supplementary Information

Additional file 1.

## Data Availability

The data are not publicly available due to the consent provided by the participant at the time of data collection. The data access, which is subject to local regulations, can be obtained upon reasonable request by contacting: Maaria Puupponen (email: maaria.puupponen@helsinki.fi), Research Program Coordinator, Clinical and Molecular Metabolism (CAMM), University of Helsinki. Upon approval, analysis needs to be performed on a user-specific local server (with protected access) and requires the applicant to sign non-disclosure and secrecy agreements.

## Abbreviations

BMI: Body mass index
CGM: Continuous glucose monitoring
CAD: Coronary artery disease
CGE_hg_: Cumulative hyperglycaemic exposure
CGE_tot_: Total cumulative glycaemic exposure
CLE_LDL_: Cumulative LDL exposure
CLE_non-HDL_: Cumulative non-HDL exposure
CLE_tg_: Cumulative triglyceride exposure
CVD: Cardiovascular disease
DCCT/EDIC: Diabetes Control and Complications Trial/Epidemiology of Diabetes Interventions and Complications study
EDC: Epidemiology of Complications study
eGFR: Estimated glomerular filtration rate
FinnDiane: Finnish Diabetic Nephropathy study
HbA_1c_: Glycated haemoglobin
HDL: High density lipoprotein
KDIGO: Kidney Disease: Improving Global Outcomes
LDL: Low density lipoprotein
