# Supplementary material for: Cumulative exposures to glycaemia and lipids are associated with coronary artery disease in type 1 diabetes: a call for action
Source: Cardiovasc Diabetol. 2025 Jun 13;24:248. doi: 10.1186/s12933-025-02803-8 (PMC12164207; doi:10.1186/s12933-025-02803-8)
Supplement: Supplementary file 1 — Additional file 1. [file 12933_2025_2803_MOESM1_ESM.docx]

**Cumulative exposures to glycaemia and lipids are associated with coronary artery disease in type 1 diabetes: a call for action**

**Supplementary material**

**Table of contents**

| **Detailed description of statistical methods** | |  | 2 |
| --- | --- | --- | --- |
| **Figure S1** | Schematic example illustration of the calculation method for cumulative glycaemic measures, total cumulative glycaemic exposure (CGE_tot_) and cumulative hyperglycaemic exposure (CGE_hg_) | | 3 |
| **Table S1** | Comparison between the individuals included in and excluded from the study | | 4 |
| **Table S2** | Baseline characteristics according to incident first-ever CAD event during follow-up, matched dataset | | 5 |
| **Table S3** | Cumulative glycaemic exposure and cumulative lipid exposure according to incident first-ever CAD event during follow-up, matched dataset | | 6 |
| **Table S4** | Competing risk regression examining the association between before baseline cumulative glycaemic exposure and first ever CAD event during follow-up | | 6 |
| **Table S5** | ­Difference in average CGE_tot_ per month before baseline, 5 years after baseline, 10 years after baseline and full follow-up, compared between CAD- and CAD+ | | 7 |
| **Table S6** | ­Difference in average CGE_hg_ per month before baseline, 5 years after baseline, 10 years after baseline and full follow-up, compared between CAD- and CAD+ | | 7 |
| **Table S7** | Competing risk regression examining the association between before baseline cumulative glycaemic exposure and first ever CAD event during follow-up, matched dataset (N = 522) | | 8 |
| **Table S8** | FinnDiane physicians and nurses at the participating study centres | | 9 |

**Detailed description of statistical methods**

In the logistic regression models CLE_LDL_ and CLE_non-HDL_ were not added to the same model due to multicollinearity. Odds ratios are reported per 100-units increase. Exceptions are CGE_hg_ and CLE_tg_ which did not fulfil the assumption of linearity and were transformed to the square root and logarithmically, respectively. Age at diabetes onset violated the linearity assumption as well and was categorized into deciles.

For the Cox proportional hazards models normal albumin excretion rate (AER) was defined as AER <30mg/24h or <20µg/min, moderate albuminuria as AER either ≥30mg/24h and ≤300mg/24h or ≥20µg/min and ≤200µg/min and severe albuminuria as AER >300mg/24h or 200µg/min. The glomerular filtration rate was estimated from serum creatinine using the CKD-EPI formula (2009 version) [1]. Hypertension was defined as systolic blood pressure above 140 mmHg, diastolic blood pressure above 90 mmHg, or use of antihypertensive medication [2]. Due to violation of the linearity assumption BMI was categorized to normal weight (BMI <25), overweight (BMI 25-30) and obese (BMI ≥30), whereas triglycerides were transformed logarithmically. The proportional hazard assumption was violated by non-HDL in the 5-year models and was therefore split into two categories by median in these models. BMI violated the same assumption in the 10-year models and was stratified. Hazard ratios are reported per 100-units for CGE_tot_ and by square root for CGE_hg_.

References

1. Levey AS, Stevens LA, Schmid CH, Zhang Y (Lucy), Castro AF, Feldman HI, et al. A New Equation to Estimate Glomerular Filtration Rate. Ann Intern Med. 2009;150:604–12.

2. Williams B, Mancia G, Spiering W, Agabiti Rosei E, Azizi M, Burnier M, et al. 2018 ESC/ESH Guidelines for the management of arterial hypertension. Eur Heart J. 2018;39:3021–104.


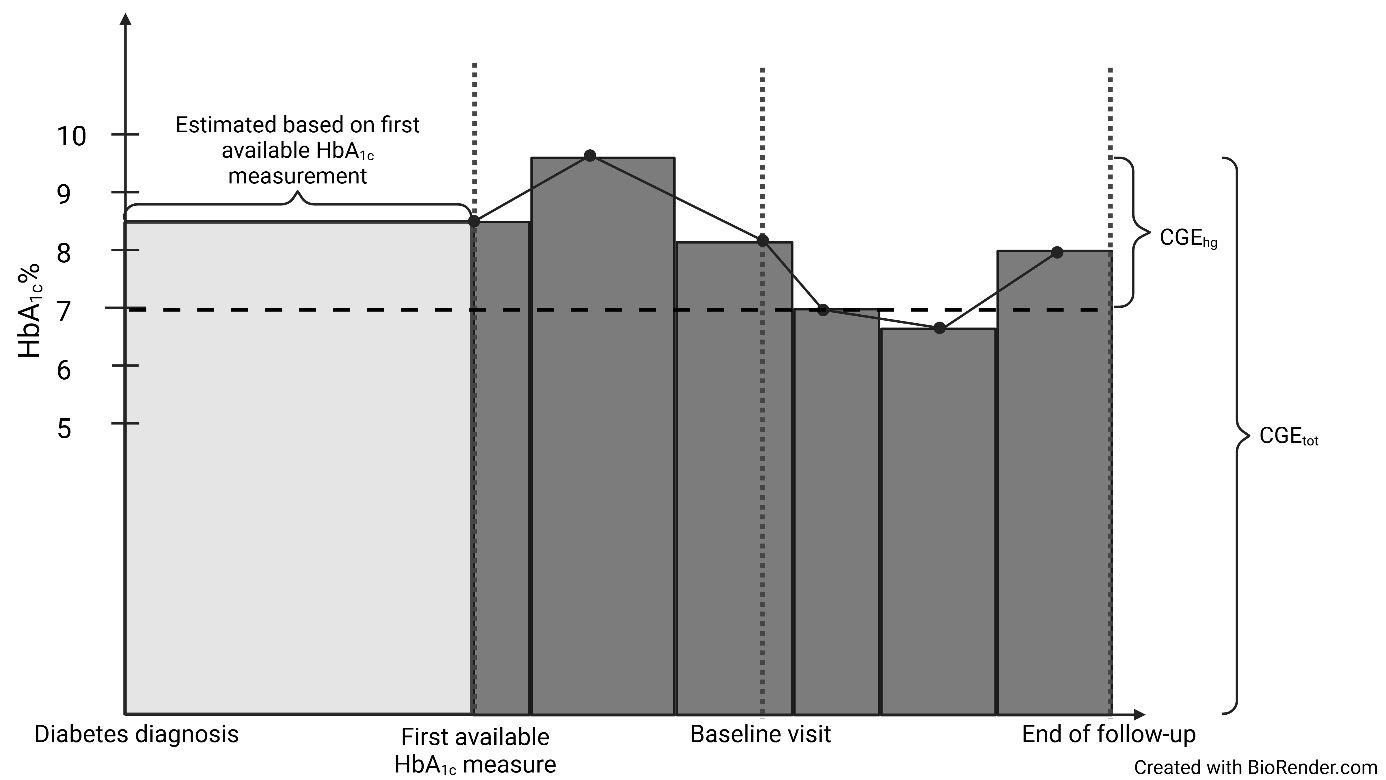


**Figure S1** Schematic example illustration of the calculation method for cumulative glycaemic measures, total cumulative glycaemic exposure (CGE_tot_) and cumulative hyperglycaemic exposure (CGE_hg_)

**Table S1** Comparison between the individuals included in and excluded from the study

| Variable | Excluded (N = 1,620) | Included (N = 3,495) | p-value |
| --- | --- | --- | --- |
| Men (%) | 54 | 50 | 0.02 |
| Age (years) | 42.6 ±12.3 | 36.9 ±11.4 | <0.001 |
| Age at onset (years) | 15.3 ±9.0 | 16.5 ±9.5 | <0.001 |
| Duration of diabetes (years) | 27.4 ±12.7 | 20.5 ±11.6 | <0.001 |
| BMI (kg/m2) | 25.2 ±4.2 | 25.2 ±3.6 | 0.85 |
| Waist/Hip-ratio | 0.90 ±0.09 | 0.69 ±0.08 | <0.001 |
| Waist/Height-ratio | 0.51 ±0.07 | 0.50 ±0.06 | <0.001 |
| SBP (mmHg) | 141 ±22 | 132 ±17 | <0.001 |
| DBP (mmHg) | 80 ±11 | 79 ±10 | 0.003 |
| HbA_1c_ (mmol/mol) | 68.0 ±15.8 | 68.5 ±16 | 0.29 |
| HbA_1c_ (%) | 8.4 ±1.4 | 8.4 ±1.5 | 0.29 |
| Antihypertensive medication (%) | 60 | 33 | <0.001 |
| Lipid-lowering medication (%) | 30 | 9 | <0.001 |
| Total cholesterol (mmol/L) | 4.9 ±1.1 | 4.9 ±0.9 | 0.37 |
| LDL (mmol/L) | 2.9 ±1.0 | 3.0 ±0.9 | <0.001 |
| HDL (mmol/L | 1.4 ±0.4 | 1.4 ±0.4 | 0.11 |
| Triglycerides (mmol/L) | 1.2 (0.8, 1.6) | 1.0 (0.8, 1.4) | <0.001 |
| ApoB (g/L) | 83.6 ±25.2 | 86.3 ±22.6 | <0.001 |
| Non–HDL (mmol/L) | 3.5 ±1.2 | 3.5 ±1.0 | 0.14 |
| eGFR (mL/min/1.73 m2) | 76 ±40 | 99±25 | <0.001 |
| Normal AER (%) | 43 | 71 | <0.001 |
| Moderate albuminuria (%) | 9 | 14 | <0.001 |
| Severe albuminuria (%) | 15 | 14 | 0.72 |
| End-stage kidney disease (%) | 33 | 0 | <0.001 |
| Ever smoker (%) | 50 | 46 | 0.002 |
| Retinal laser treatment (%) | 55 | 27 | <0.001 |
| Any cardiovascular disease before baseline (%) | 24 | 0 | <0.001 |

%; Mean ±SD; Median (IQR)

**Table S2** Baseline characteristics according to incident first-ever CAD event during follow-up, matched dataset

| Variable | N | CAD -, N = 1,068^a^ | CAD +, N = 534^a^ | p-value |
| --- | --- | --- | --- | --- |
| Men (%) | 1,602 | 49 | 54 | 0.050 |
| Age (years) | 1,602 | 41.5 ±11.1 | 44.7 ±10.8 | <0.001 |
| Age at onset (years) | 1,602 | 14.0 ±8.7 | 16.1 ±9.5 | <0.001 |
| Duration of diabetes (years) | 1,602 | 27.5 ±10.7 | 28.7 ±11.0 | 0.038 |
| BMI (kg/m2) | 1,590 | 25.2 ±3.6 | 25.8 ±3.6 | 0.002 |
| Waist/Hip-ratio | 1,554 | 0.86 ±0.08 | 0.89 ±0.09 | <0.001 |
| Waist/Height-ratio | 1,553 | 0.50 ±0.06 | 0.52 ±0.06 | <0.001 |
| SBP (mmHg) | 1,587 | 134 ±17 | 141 ±18 | <0.001 |
| DBP (mmHg) | 1,587 | 79 ±9 | 81 ±10 | <0.001 |
| HbA_1c_ (mmol/mol) | 1,602 | 67.2 ±14.4 | 72.7 ±15.4 | <0.001 |
| HbA_1c_ (%) | 1,602 | 8.3 ±1.3 | 8.8 ±1.4 | <0.001 |
| Antihypertensive medication (%) | 1,590 | 40 | 58 | <0.001 |
| Lipid-lowering medication (%) | 1,594 | 11 | 20 | <0.001 |
| Total cholesterol (mmol/L) | 1,594 | 4.9 ±0.9 | 5.3 ±1.0 | <0.001 |
| LDL (mmol/L) | 1,594 | 3.1 ±0.9 | 3.4 ±0.9 | <0.001 |
| HDL (mmol/L | 1,594 | 1.4 ±0.4 | 1.3 ±0.4 | <0.001 |
| Triglycerides (mmol/L) | 1,594 | 0.9 (0.7, 1.3) | 1.2 (0.9, 1.7) | <0.001 |
| ApoB (g/L) | 1,570 | 84.7 ±21.8 | 96.1 ±22.7 | <0.001 |
| Non–HDL (mmol/L) | 1,594 | 3.5 ±1.0 | 4.0 ±1.1 | <0.001 |
| eGFR (mL/min/1.73 m2) | 1,602 | 94.3 ±23.9 | 84.5 ±28.6 | <0.001 |
| eGDR (mg/kg/min) | 1,549 | 6.1 (4.5, 8.4) | 4.8 (3.5, 6.7) | <0.001 |
| Normal AER (%) | 1,602 | 67 | 49 | <0.001 |
| Moderate albuminuria (%) | 1,602 | 18 | 18 | 0.8 |
| Severe albuminuria (%) | 1,602 | 16 | 33 | <0.001 |
| Ever smoker (%) | 1,523 | 46 | 50 | 0.14 |
| Retinal laser treatment (%) | 1,588 | 37 | 53 | <0.001 |

^a^%; Mean ±SD; Median (IQR)

**Table S3** Cumulative glycaemic exposure and cumulative lipid exposure according to incident first-ever CAD event during follow-up, matched dataset

| Variable | N | CAD-, N = 1,068^a^ | CAD+, N = 534^a^ | p-value |
| --- | --- | --- | --- | --- |
| Diabetes duration at end of follow-up | 1,602 | 45.35 ±11.07 | 41.03 ±10.91 | <0.001 |
| Follow-up time (years) | 1,602 | 19.61 (16.30, 21.61) | 13.10 (7.83, 17.02) | <0.001 |
| Time from diagnosis to first HbA_1c_ measurement (years) | 1,602 | 26.51 (18.92, 33.20) | 26.48 (18.92, 34.54) | 0.4 |
| Number of HbA_1c_ measurements | 1,602 | 27.00 (15.00, 39.00) | 24.00 (13.00, 38.75) | 0.071 |
| CGE_tot_ | 1,602 | 4,523.38 ±1,182.57 | 4,349.04 ±1,193.63 | 0.006 |
| CGE_hg_ | 1,602 | 667.53 (334.92, 1,038.94) | 843.57 (509.16, 1,268.72) | <0.001 |
| Time from diagnosis to first lipid measurement (years) | 1,347 | 27.37 (19.19, 33.17) | 27.31 (20.47, 34.90) | 0.2 |
| Number of lipid measurements | 1,347 | 11.00 (7.00, 17.00) | 10.00 (7.00, 15.00) | 0.11 |
| CLE_LDL_ | 1,347 | 1,552.27 ±536.04 | 1,582.28 ±550.96 | 0.3 |
| CLE_tg_ | 1,347 | 500.99 (382.79, 690.13) | 565.67 (403.58, 824.12) | <0.001 |
| CLE_non-HDL_ | 1,347 | 1,794.67 ±596.10 | 1,859.71 ±625.30 | 0.072 |

^a^Median (IQR); Mean ±SD

**Table S4** Competing risk regression examining the association between before baseline cumulative glycaemic exposure and first ever CAD event during follow-up

| Competing risk model, full follow-up, N = 174 | Variable | HR (CI 95%) | p-value |
| --- | --- | --- | --- |
| Model 4 | CGE_tot_ | 1.03 (1.00-1.07) | 0.075 |
| Model 4 | CGE_hg_ | 1.02 (1.00-1.04) | 0.024 |

Model 4 adjusted for unmodifiable risk factors (sex, age, and diabetes duration), lipids (non-HDL cholesterol and triglycerides), hypertension, lipid-lowering medication, obesity, and kidney disease and further for CGE/month after baseline

^a^CGE_tot_ is reported per 100-units, CGE**_hg_** by square root

**Table S5** ­Difference in average CGE_tot_ per month before baseline, 5 years after baseline, 10 years after baseline and full follow-up, compared between CAD- and CAD+

| Variable | CAD-, N = 947^a^ | CAD+, N = 174^a^ | p-value |
| --- | --- | --- | --- |
| Before baseline CGE_tot_/month | 8.61 ±1.48 | 9.17 ±1.60 | <0.001 |
| Δ5 years | -0.25 ±1.26 | -0.42 ±1.47 | 0.13 |
| Δ10 years | -0.30 ±1.27 | -0.51 ±1.52 | 0.092 |
| Δ follow-up | -0.36 ±1.31 | -0.53 ±1.57 | 0.2 |

^a^Mean ±SD

**Table S6** ­Difference in average CGE_hg_ per month before baseline, 5 years after baseline, 10 years after baseline and full follow-up, compared between CAD- and CAD+.

| Variable | CAD-, N = 947^a^ | CAD+, N = 174^b^ | p-value |
| --- | --- | --- | --- |
| Before baseline CGE_hg_/month | 1.46 (0.63, 2.31) | 2.00 (1.23, 3.03) | <0.001 |
| Δ5 years | -0.24 ±1.16 | -0.43 ±1.43 | 0.095 |
| Δ10 years | -0.29 ±1.18 | -0.51 ±1.47 | 0.064 |
| Δ follow-up | -0.35 ±1.20 | -0.54 ±1.51 | 0.13 |

^a^Median (IQR); Mean ±SD

**Table S7** Competing risk regression examining the association between before baseline cumulative glycaemic exposure and first ever CAD event during follow-up, matched dataset (N = 522)

| Competing risk model, 5 years, N = 35 | Variable | HR (CI 95%)^a^ | p-value |
| --- | --- | --- | --- |
| Model 1 | CGE_tot_ | 1.08 (1.03-1.13) | 0.002 |
| Model 2 | CGE_tot_ | 1.07 (1.02-1.12) | 0.010 |
| Model 3 | CGE_tot_ | 1.06 (1.00-1.11) | 0.036 |
| Competing risk model, 5 years, N = 35 | Variable | HR (CI 95%) | p-value |
| Model 1 | CGE_hg_ | 1.06 (1.02-1.09) | 0.001 |
| Model 2 | CGE_hg_ | 1.05 (1.02-1.08) | 0.004 |
| Model 3 | CGE_hg_ | 1.04 (1.01-1.08) | 0.016 |
| Competing risk model, 10 years, N = 75 | Variable | HR (CI 95%) | p-value |
| Model 1 | CGE_tot_ | 1.06 (1.02-1.10) | 0.001 |
| Model 2 | CGE_tot_ | 1.06 (1.02-1.10) | 0.004 |
| Model 3 | CGE_tot_ | 1.05 (1.01-1.09) | 0.017 |
| Competing risk model, 10 years, N = 75 | Variable | HR (CI 95%) | p-value |
| Model 1 | CGE_hg_ | 1.04 (1.02-1.07) | <0.001 |
| Model 2 | CGE_hg_ | 1.04 (1.01-1.06) | 0.002 |
| Model 3 | CGE_hg_ | 1.03 (1.01-1.06) | 0.007 |
| Competing risk model, full follow-up, N = 174 | Variable | HR (CI 95%) | p-value |
| Model 1 | CGE_tot_ | 1.08 (1.05-1.10) | <0.001 |
| Model 2 | CGE_tot_ | 1.07 (1.04-1.10) | <0.001 |
| Model 3 | CGE_tot_ | 1.07 (1.04-1.10) | <0.001 |
| Competing risk model, full follow-up, N = 174 | Variable | HR (CI 95%) | p-value |
| Model 1 | CGE_hg_ | 1.05 (1.03-1.06) | <0.001 |
| Model 2 | CGE_hg_ | 1.04 (1.03-1.06) | <0.001 |
| Model 3 | CGE_hg_ | 1.04 (1.02-1.06) | <0.001 |

Model 1 adjusted for unmodifiable risk factors sex, age, and diabetes duration; model 2 adjusted for unmodifiable risk factors and lipids: non-HDL cholesterol and triglycerides; model 3 adjusted for unmodifiable risk factors, lipids and hypertension, lipid-lowering medication, obesity, and kidney disease

^a^CGE_tot_ is reported per 100-units, CGE_hg_ by square root

**Table S8** FinnDiane physicians and nurses at the participating study centres

| Anjalankoski Health Center | S.Koivula, T.Uggeldahl |
| --- | --- |
| Central Finland Central Hospital, Jyväskylä | T.Forslund, A.Halonen, A.Koistinen, P.Koskiaho, M.Laukkanen, J.Saltevo, M.Tiihonen |
| Central Hospital of Åland Islands, Mariehamn | M.Forsen, H.Granlund, A.-C.Jonsson, B.Nyroos |
| Central Hospital of Kanta-Häme, Hämeenlinna | P.Kinnunen, A.Orvola, T.Salonen, A.Vähänen |
| Central Hospital of Kymenlaakso, Kotka | R.Paldanius, M.Riihelä, L.Ryysy |
| Central Hospital of Länsi-Pohja, Kemi | H.Laukkanen, P.Nyländen, A.Sademies |
| Central Ostrobothnian Hospital District, Kokkola | S.Anderson, B.Asplund, U.Byskata, P.Liedes, M.Kuusela, T.Virkkala |
| City of Espoo Health Center: |  |
| Espoonlahti | A.Nikkola, E.Ritola |
| Tapiola | M.Niska, H.Saarinen |
| Samaria | E.Oukko-Ruponen, T.Virtanen |
| Viherlaakso | A.Lyytinen |
| City of Helsinki Health Center: |  |
| Puistola | H.Kari, T.Simonen |
| Suutarila | A.Kaprio, J.Kärkkäinen, B.Rantaeskola |
| Töölö | P.Kääriäinen, J.Haaga, A-L.Pietiläinen |
| City of Hyvinkää Health Center | S.Klemetti, T.Nyandoto, E.Rontu, S.Satuli-Autere |
| City of Vantaa Health Center: |  |
| Korso | R.Toivonen, H.Virtanen |
| Länsimäki | R.Ahonen, M.Ivaska-Suomela, A.Jauhiainen |
| Martinlaakso | M.Laine, T.Pellonpää, R.Puranen |
| Myyrmäki | A.Airas, J.Laakso, K.Rautavaara |
| Rekola | M.Erola, E.Jatkola |
| Tikkurila | R.Lönnblad, A.Malm, J.Mäkelä, E.Rautamo |
| Heinola Health Center | P.Hentunen, J.Lagerstam |
| Helsinki University Central Hospital, Department of Medicine, Division of Nephrology | A.Ahola, M.Feodoroff, D.Gordin, O.Heikkilä, K.Hietala, M.Korolainen J.Kytö, S.Lindh, K.Pettersson-Fernholm, A.Sandelin, L.Thorn, J.Tuomikangas, T.Vesisenaho, J.Wadén |
| Herttoniemi Hospital, Helsinki | V.Sipilä |
| Hospital of Lounais-Häme, Forssa | T.Kalliomäki, J.Koskelainen, R.Nikkanen, N.Savolainen, H.Sulonen, E.Valtonen |
| Hyvinkää Hospital | L. Norvio, A. Hämäläinen |
| Iisalmi Hospital | E.Toivanen |
| Jokilaakso Hospital, Jämsä | A.Parta, I.Pirttiniemi |
| Jorvi Hospital, Helsinki University Central Hospital | S.Aranko, S.Ervasti, R.Kauppinen-Mäkelin, A.Kuusisto, T.Leppälä, K.Nikkilä, L.Pekkonen |
| Jyväskylä Health Center, Kyllö | K.Nuorva, M.Tiihonen |
| Kainuu Central Hospital, Kajaani | S.Jokelainen, K.Kananen, M.Karjalainen, P.Kemppainen, A-M.Mankinen, A.Reponen, M.Sankari |
| Kerava Health Center | H.Stuckey, P.Suominen |
| Kirkkonummi Health Center | A.Lappalainen, M.Liimatainen, J.Santaholma |
| Kivelä Hospital, Helsinki | A.Aimolahti, E.Huovinen |
| Koskela Hospital, Helsinki | V.Ilkka, M.Lehtimäki |
| Kotka Health Center | E.Pälikkö-Kontinen, A.Vanhanen |
| Kouvola Health Center | E.Koskinen, T.Siitonen |
| Kuopio University Hospital | E.Huttunen, R.Ikäheimo, P.Karhapää, P.Kekäläinen, M.Laakso, T.Lakka, E.Lampainen, L.Moilanen, L.Niskanen, U.Tuovinen, I.Vauhkonen, E.Voutilainen |
| Kuusamo Health Center | T.Kääriäinen, E.Isopoussu |
| Kuusankoski Hospital | E.Kilkki, I.Koskinen, L.Riihelä |
| Laakso Hospital, Helsinki | T.Meriläinen, P.Poukka, R.Savolainen, N.Uhlenius |
| Lahti City Hospital | A.Mäkelä, M.Tanner |
| Lapland Central Hospital, Rovaniemi | L.Hyvärinen, K.Lampela, S.Pöykkö, T.Rompasaari, S.Severinkangas, T.Tulokas |
| Lappeenranta Health Center | P. Erola, L. Härkönen, P.Linkola, I.Pulli, E.Repo |
| Lohja Hospital | T.Granlund, K.Hietanen, M.Porrassalmi, M.Saari, T.Salonen, M.Tiikkainen, |
| Länsi-Uusimaa Hospital, Tammisaari | I.-M.Jousmaa, J.Rinne |
| Loimaa Health Center | A.Mäkelä, P.Eloranta |
| Malmi Hospital, Helsinki | H.Lanki, S.Moilanen, M.Tilly-Kiesi |
| Mikkeli Central Hospital | A.Gynther, R.Manninen, P.Nironen, M.Salminen, T.Vänttinen |
| Mänttä Regional Hospital | I.Pirttiniemi, A-M.Hänninen |
| North Karelian Hospital, Joensuu | U-M.Henttula, P.Kekäläinen, M.Pietarinen, A.Rissanen, M.Voutilainen |
| Nurmijärvi Health Center | A.Burgos, K.Urtamo |
| Oulaskangas Hospital, Oulainen | E.Jokelainen, P-L.Jylkkä, E.Kaarlela, J.Vuolaspuro |
| Oulu Health Center | L.Hiltunen, R.Häkkinen, S.Keinänen-Kiukaanniemi |
| Oulu University Hospital | R.Ikäheimo |
| Päijät-Häme Central Hospital | H.Haapamäki, A.Helanterä, S.Hämäläinen, V.Ilvesmäki, H.Miettinen |
| Palokka Health Center | P.Sopanen, L.Welling |
| Pieksämäki Hospital | V.Sevtsenko, M.Tamminen |
| Pietarsaari Hospital | M-L.Holmbäck, B.Isomaa, L.Sarelin |
| Pori City Hospital | P.Ahonen, P.Merisalo, E.Muurinen, K.Sävelä |
| Porvoo Hospital | M.Kallio, B.Rask, S.Rämö |
| Raahe Hospital | A.Holma, M.Honkala, A.Tuomivaara, R.Vainionpää |
| Rauma Hospital | K.Laine, K.Saarinen, T.Salminen |
| Riihimäki Hospital | P.Aalto, E.Immonen, L.Juurinen |
| Salo Hospital | A.Alanko, J.Lapinleimu, P.Rautio, M.Virtanen |
| Satakunta Central Hospital, Pori | M.Asola, M.Juhola, P.Kunelius, M.-L.Lahdenmäki, P.Pääkkönen, M.Rautavirta |
| Savonlinna Central Hospital | T.Pulli, P.Sallinen, M.Taskinen, E.Tolvanen, T.Tuominen, H.Valtonen, A.Vartia, S-L.Viitanen |
| Seinäjoki Central Hospital | O.Antila, E.Korpi-Hyövälti, T.Latvala, E.Leijala, T.Leikkari, M.Punkari N.Rantamäki, H.Vähävuori |
| South Karelia Central Hospital, Lappeenranta | T.Ensala, E.Hussi, R.Härkönen, U.Nyholm, J.Toivanen |
| Tampere Health Center | A.Vaden, P.Alarotu, E.Kujansuu, H.Kirkkopelto-Jokinen, M.Helin, S.Gummerus, L.Calonius, T.Niskanen, T.Kaitala, T.Vatanen |
| Tampere University Hospital | I.Ala-Houhala, R.Kannisto, T.Kuningas, P.Lampinen, M.Määttä, H.Oksala, T.Oksanen, A.Putila, H.Saha, K.Salonen, H.Tauriainen, S.Tulokas |
| Tiirismaa Health Center, Hollola | T.Kivelä, L.Petlin, L.Savolainen |
| Turku Health Center | A.Artukka, I.Hämäläinen, L.Lehtinen, E.Pyysalo, H.Virtamo, M.Viinikkala, M.Vähätalo |
| Turku University Central Hospital | K.Breitholz, R.Eskola, K.Metsärinne, U.Pietilä, P.Saarinen, R.Tuominen, S.Äyräpää |
| Vaajakoski Health Center | K.Mäkinen, P.Sopanen |
| Valkeakoski Regional Hospital | S.Ojanen, E.Valtonen, H.Ylönen, M.Rautiainen, T.Immonen |
| Vammala Regional Hospital | I.Isomäki, R.Kroneld, L.Mustaniemi, M.Tapiolinna-Mäkelä |
| Vasa Central Hospital | S.Bergkulla, U.Hautamäki, V-A.Myllyniemi, I.Rusk |
